# A CT-Based Radiomics Nomogram to Predict Complete Ablation of Pulmonary Malignancy: A Multicenter Study

**DOI:** 10.3389/fonc.2022.841678

**Published:** 2022-02-10

**Authors:** Guozheng Zhang, Hong Yang, Xisong Zhu, Jun Luo, Jiaping Zheng, Yining Xu, Yifeng Zheng, Yuguo Wei, Zubing Mei, Guoliang Shao

**Affiliations:** ^1^ Department of Radiology, The Quzhou Affiliated Hospital of Wenzhou Medical University (Quzhou People’s Hospital), Quzhou, China; ^2^ Department of Radiology, Cancer Hospital of the University of Chinese Academy of Sciences (Zhejiang Cancer Hospital), Institute of Basic Medicine and Cancer (IBMC), Chinese Academy of Sciences, Hangzhou, China; ^3^ Department of Interventional Radiology, Cancer Hospital of the University of Chinese Academy of Sciences (Zhejiang Cancer Hospital), Institute of Basic Medicine and Cancer (IBMC), Chinese Academy of Sciences, Hangzhou, China; ^4^ Department of Radiology, Huzhou Central Hospital, Huzhou, China; ^5^ Precision Health Institution, General Electric (GE) Healthcare, Hangzhou, China; ^6^ Department of Anorectal Surgery, Shuguang Hospital Affiliated to Shanghai University of Traditional Chinese Medicine, Shanghai, China; ^7^ Anorectal Disease Institute of Shuguang Hospital, Shanghai, China

**Keywords:** pulmonary malignancy, ablation, nomogram, radiomics, prediction model

## Abstract

**Objective:**

Thermal ablation is a minimally invasive procedure for the treatment of pulmonary malignancy, but the intraoperative measure of complete ablation of the tumor is mainly based on the subjective judgment of clinicians without quantitative criteria. This study aimed to develop and validate an intraoperative computed tomography (CT)-based radiomic nomogram to predict complete ablation of pulmonary malignancy.

**Methods:**

This study enrolled 104 individual lesions from 92 patients with primary or metastatic pulmonary malignancies, which were randomly divided into training cohort (n=74) and verification cohort (n=30). Radiomics features were extracted from the original CT images when the study clinicians determined the completion of the ablation surgery. Minimum redundancy maximum relevance (mRMR) and least absolute shrinkage and selection operator (LASSO) were adopted for the dimensionality reduction of high-dimensional data and feature selection. The prediction model was developed based on the radiomics signature combined with the independent clinical predictors by multiple logistic regression analysis. The area under the curve (AUC), accuracy, sensitivity, and specificity were calculated. Receiver operating characteristic (ROC) curves and calibration curves were used to evaluate the predictive performance of the model. Decision curve analysis (DCA) was applied to estimate the clinical usefulness and net benefit of the nomogram for decision making.

**Results:**

Thirteen CT features were selected to construct radiomics prediction model, which exhibits good predictive performance for determination of complete ablation of pulmonary malignancy. The AUCs of a CT-based radiomics nomogram that integrated the radiomics signature and the clinical predictors were 0.88 (95% CI 0.80-0.96) in the training cohort and 0.87 (95% CI: 0.71–1.00) in the validation cohort, respectively. The radiomics nomogram was well calibrated in both the training and validation cohorts, and it was highly consistent with complete tumor ablation. DCA indicated that the nomogram was clinically useful.

**Conclusion:**

A CT-based radiomics nomogram has good predictive value for determination of complete ablation of pulmonary malignancy intraoperatively, which can assist in decision-making.

## Introduction

Primary lung cancer is the second most common malignant tumor and the leading cause of cancer-related death worldwide (1). Surgical resection is recommended for early-stage primary lung cancer (stage I and II), and inoperable patients can be treated with stereotactic radiotherapy and systemic chemotherapy (2, 3). Thermal ablation is a minimally invasive method for local treatment of pulmonary malignancies, which has been widely used in recent years for the treatment of primary and metastatic lung cancer (4–6). The overall survival rate of non-small cell lung cancer (NSCLC) stage IA patients treated with thermal ablation is not lower than that of stereotactic radiotherapy (7). This technique has been gradually accepted by clinicians and patients because of its effectiveness, minimal complications, and high rate of local control (8–10).

Thermal ablation is an *in situ* treatment option (11), the efficacy of which could not be determined by intraoperative pathological findings without postoperative follow-up monitoring for residual or recurrent lesions. Therefore, it is important to intraoperatively evaluate whether complete ablation of the pulmonary malignancy was achieved (12). Current guidelines recommend that a 1-cm ground-glass shadow around the lesion after thermal ablation is used to judge complete ablation of the lesion (13). However, there are difficulties associated with this approach. First, ground-glass shadow comprises pathologically necrotic and hyperemic exudate of the lung tissue (14, 15), and tumor cells may remain in the hyperemic exudate area. Second, bleeding caused by intraoperative puncture also manifests as ground-glass shadow, which obscures the ground-glass shadow produced by thermal ablation. It is sometimes difficult to determine the intraoperative complete ablation of the tumor by macroscopic observation. Intraoperative computed tomography (CT) enhancement can be used to evaluate the efficacy of ablation, but the use of contrast agents increases the risk of iodine allergy. Moreover, it is difficult to visually identify smaller lesions after enhanced scans.

Radiomics based on artificial intelligence is a noninvasive, efficient, and reliable method, which has attracted increasing attention. It involves the high-throughput extraction of quantitative imaging features and screening of regions of interest, as well as the establishment of a radiomics prediction model (16–21). To our knowledge, no study has been reported on the prediction of the immediate outcome of thermal ablation for pulmonary malignancies based on CT radiometric features.

Therefore, we attempted to extract quantitative imaging features from intraoperative CT images, then developed a CT-based radiomics nomogram by incorporating the optimal configuration of radiomics signatures and potential independent clinical predictors to assess the immediate efficacy of thermal ablation for pulmonary malignancies. We aimed to provide a reference for operators to make decisions during surgery, such as whether it is necessary to increase the ablation parameters (power and time) or adjust the ablation needle, to ensure complete ablation of pulmonary malignancies.

## Methods

This retrospective study was approved by the ethics committees of Cancer Hospital of the University of Chinese Academy of Sciences and Huzhou Central Hospital. Requirement for informed consent was waived. This study adhered to the Transparent Reporting of a Multivariable Prediction Model for Individual Prognosis or Diagnosis (TRIPOD) reporting guideline for prediction model development and validation (22) (**Supplementary TRIPOD Checklist**).

### Study Participants

The clinical data of patients undergoing thermal ablation for pulmonary malignancies collected from Cancer Hospital of the University of Chinese Academy of Sciences and Huzhou Central Hospital between May 2008 and November 2019 were analyzed. The inclusion criteria were as follows: 1) pulmonary malignancy confirmed by puncture biopsy or clinical diagnosis of metastatic pulmonary malignancy; 2) enhanced chest CT examination performed before treatment; 3) regular plain chest scan and enhanced CT examination performed after treatment; 4) and successful ablation of pulmonary malignancy. The exclusion criteria included the following: 1) extensive respiratory artifacts on CT images; 2) incomplete clinical and imaging data; 3) and/or loss to follow-up for various reasons. The collected clinical data were carefully checked and verified by the researchers. Finally,104 lesions in 92 patients (63 males, 29 women; mean age 60.24 ± 10.20 years; range,35-80 years) fulfilled the criteria and were randomly assigned to training cohort (n=74) and validation cohort (n=30).

The clinical data included patient age and sex; imaging data included the largest transverse and longitudinal tumor diameters, tumor morphology, tumor location, bronchus and blood vessels with the largest diameter around the tumor within 1 cm, and the distance between the tumor and the pleura, as shown in **Table 1** and **Supplementary Table 1**.

**Table 1:** Characteristics of patients in the training and validation cohorts.

		Training Cohort			Validation Cohort			
		Complete ablation (n = 47)	Incomplete ablation (n = 27)	*p*	Complete ablation (n = 19)	Incomplete ablation (n = 11)	*p*
Age, years (mean ± SD)		61.1 (10.9)	57.6 (9.2)	0.161	61.6 (9.7)	63.1 (9.3)	0.677
Gender (%)							
Male		34 (72.3)	24 (88.9)		8 (42.1)	8 (72.7)	
Female		13 (27.7)	3 (11.1)	0.172	11 (57.9)	3 (27.3)	0.142
Treatment options							
Radio-frequency ablation		39 (83.0)	23 (85.2)		18 (94.7)	10 (90.9)	
Microwave ablation		8 (17.0)	4 (14.8)	1.00	1 (5.3)	1 (9.1)	1.00
Nodule Shape							
Class round		39 (83.0)	23 (85.2)		16 (84.2)	6 (54.5)	
Irregularly shaped		8 (17.0)	4 (14.8)	1.00	3 (15.8)	5 (45.5)	0.180
LD(mean ± SD)		15.9 ± 5.6	20.2 ± 7.5	0.006*	16.0 ± 4.9	29.9 ± 12.6	<0.01*
TD(mean ± SD)		12.6 ± 4.9	16.8 ± 6.0	0.001*	13.0 ± 4.4	20.6 ± 8.6	0.001*
LD/TD		1.3 (0.3)	1.2 (0.2)	0.114	1.2 (0.2)	1.5 (0.4)	0.028*
Bronchial diameter							
1mm		7 (14.9)	4 (14.8)	1.00	5 (26.3)	1 (9.1)	0.507
2mm		23 (48.9)	3 (11.1)	0.002*	7 (36.8)	2 (18.2)	0.508
3mm		11 (23.4)	9 (33.3)	0.513	6 (31.6)	3 (27.3)	1.00
4mm		4 (8.5)	4 (14.8)	0.651	1 (5.3)	2 (18.2)	0.613
5mm		2 (4.3)	5 (18.5)	0.108	0 (0.0)	2 (18.2)	0.244
6mm		0 (0.0)	2 (7.4)	0.251	0 (0.0)	1 (9.1)	0.778
Blood vessel diameter							
1mm		20 (42.6)	9 (33.3)	0.593	9 (47.4)	1 (9.1)	0.082
2mm		17 (36.2)	9 (33.3)	1.00	7 (36.8)	4 (36.4)	1.000
3mm		8 (17.0)	5 (18.5)	1.00	3 (15.8)	4 (36.4)	0.403
4mm		2 (4.3)	4 (14.8)	0.246	0 (0.0)	2 (18.2)	0.244
Tumor location							
The right lung	superior lobe	11 (23.4)	9 (33.3)	0.513	4 (21.1)	2 (18.2)	1.000
	middle lobe	6 (12.8)	1 (3.7)	0.384	2 (10.5)	0 (0.0)	0.723
	inferior lobe	9 (19.1)	6 (22.2)	0.987	5 (26.3)	3 (27.3)	1.000
The left lung	superior lobe	6 (12.8)	6 (22.2)	0.462	3 (15.8)	3 (27.3)	0.776
	inferior lobe	15 (31.9)	5 (18.5)	0.328	5 (26.3)	3 (27.3)	1.000
Distance from nodule to pleura(mean ± SD)		14.1 (10.6)	12.5 (10.3)	0.532	11.1 (8.9)	10.5 (8.0)	0.846

D, nodule shortest diameter; LD, nodule longest diameter; BL2, the largest vessel diameter within 1cm around the nodules was 2 mm; LD/TD, ratio of long diameter to short diameter; Bronchial diameter, the largest vascular diameter within 1cm around the nodule; Blood vessel diameter, the largest diameter bronchus within 1cm around the nodule. In Bold: *statistically significant (P < 0.05).

The patients were followed up for 6–38 months, with a median follow-up of 21 months. When patients had two or three lesions in the lung, each individual lesion was followed. In accordance with image-guided tumor ablation recommendations in 2014 (13), complete ablation was defined as complete disappearance of the lesion or no enhancement on enhanced scan lasting for at least 3 months; otherwise, the ablation was considered incomplete.

### Radiofrequency and Microwave Ablation

Prior to ablation, plain CT scans were performed first. The scanning position of the patient was determined according to the location of the lesion and supine position was preferred to facilitate anesthesia. Under general anesthesia with electrocardiogram monitoring, the radiofrequency or microwave ablation instrument was adjusted. CT scans were performed to locate the lesions and the site was marked on the skin surface. The surgical area was disinfected, and local anesthesia was provided at the puncture site with 5 ml 1% lidocaine hydrochloride. Under CT guidance, an ablation needle was inserted into the lesion site. The position of the needle tip was adjusted to find the optimal position. A tumor ablation instrument (KY-2000, Kangyou Medical Instruments Company, Nanjing, China) with an operating frequency of 2,450 MHz&an output power of 0~150 W and an RF ablation system (CTRF-220, Tyco Healthcare, Puritan Bennett, California, US) with an operating frequency of 480 kHz and an output power of 0~200W were used (**Supplementary Table 2**). Following manufacturer’s instructions, appropriate parameters (power and time) were selected to determine the success of thermal ablation when more than 1 cm ground-glass shadows around the tumor lesions were shown. The needle passage was then ablated to prevent tumor implantation along the needle track. The ablation needle was removed, and the surgery finished. Electrocardiogram monitoring was performed continuously over 24 h. Hemostasis and other symptomatic treatments were also carried out. Plain chest CT examinations were performed immediately post-operation to detect any pneumothorax, bleeding, or other complications. Patients were evaluated 1 month postoperatively and every 3 months by enhanced CT scans to evaluate the treatment effect after ablation.

### CT Image Acquisition and Imaging Evaluation

Thermal ablation was performed under the guidance of CT scan. The detailed scan and reconstruction parameters are listed in **Supplementary Table 3**. CT scans were performed during the operation. When a more than 1-cm ground-glass shadow around the lesion was observed, it was judged as complete ablation of the lesion. The original CT images were collected at this time.

Two reviewers who had 5 years (Hong Yang) and 12 years (Guozheng Zhang) of experience with chest CT image interpretation reviewed all CT images to evaluate the following characteristics of each pulmonary malignancy: (a) tumor location and number; (b) the long and short diameters of the maximum tumor layer;(c) maximum diameters of vessels and bronchi around the tumor (defined as maximum diameters of bronchi and vessels within 1 cm from the tumor); (d) shape of the tumor lesion (defined as class round or irregular); (f) and distance between tumor and pleura (defined as the shortest distance between tumor and pleura). Both radiologists knew that thermal ablation of the pulmonary malignancy had been performed, but they had no prior information regarding whether a particular tumor had been completely ablated. For 4 weeks, the CT images were reviewed daily; disagreements were resolved by a senior radiologist (with 15 years of chest tumor imaging experience).

### Segmentation of the Region of Interest and Radiomics Feature Extraction

#### ROI Segmentation

Manual delineation of the ROI was performed on selected CT images of each patient’s pulmonary lesion using ITK-SNAP software (Version 3.4.0, http://www.itksnap.org/) and 3-dimention volume of interest (VOI) was synthesized. The VOI included the lesion, 1-cm ground-glass shadows around the tumor lesions after thermal ablation and regions depicted in a layer-by-layer manner along the ground-glass shadows. Adjacent aorta, ribs, and pneumothorax were excluded. All target lesions were delineated by 2 radiologists. Disagreements were resolved by a senior radiologist.

#### Radiomics Feature Extraction

**Figure 1:**
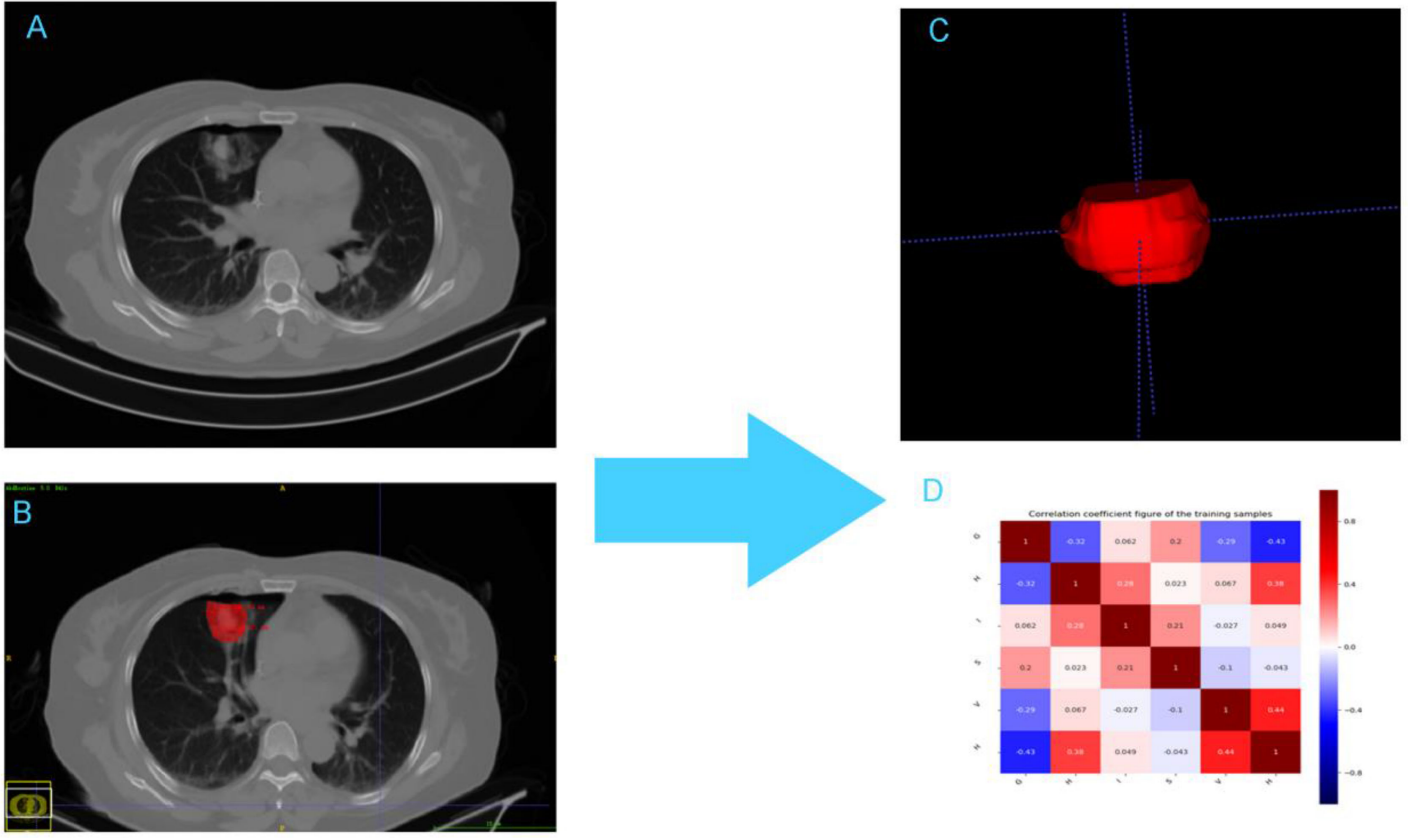
The framework for the radiomic workflow. **(A)** CT acquisition **(B)** Ablation lesion segmention **(C)** Feature extraction **(D)** Heatmap of the correlation of the radiomic features.

AK software (artificial intelligence suite V3.0.0.R, GE Healthcare) was used to extract 396 radiomics features (e.g., first-order features, second-order features, and morphological parameters) from the VOI of CT images in 104 ablation foci of pulmonary malignancies. First-order features generally described the distribution of individual voxels, regardless of the spatial relationship among voxels. Second-order features generally comprised “texture” features, including gray level co-occurrence matrix (GLCM) and gray level run-length matrix features (GLRLM). They described the surface appearance and the spatial distribution of voxels (21, 23, 24). Prior to feature dimension reduction, each feature value for all patients was normalized using a Z-score as follows: Z = (x-μ)/σ, where x was the value of the feature, μ was the mean value of the feature for all patients in the cohort, and σ was the corresponding standard deviation. Z-score was used to remove the unit limitation for each feature before machine learning classification. **Figure 1** shows the radiomics workflow and study flow chart.

### Agreements Within and Between Observers

The intraclass correlation coefficient (ICC) was used to evaluate consistency between observers. CT image data in 45 thermal ablation foci of pulmonary malignancies treated at Cancer Hospital of the University of Chinese Academy of Sciences were randomly selected from the study cohort. After 3 months, intra-observer segmentation was performed by a radiologist with 12 years of chest imaging experience and inter-observer segmentation reproducibility was performed by another radiologist with 5 years chest imaging experience. Independent samples t-tests were used for statistical comparisons (p-value < 0.05). When the ICC was greater than 0.75, the consistency was considered good (25).

### Model Construction and Validation

Minimum redundancy maximum relevance (mRMR) and least absolute shrinkage and selection operator (LASSO) were used to select CT feature from the training cohort. First, high-throughput CT image processing was performed using mRMR method. The top 20 most powerful features with the greatest correlation with complete ablation and the least redundancy were selected. The LASSO algorithm with ten-fold cross validation was used to select the optimal subset of predictive features and evaluate the corresponding feature coefficients. The radiomics feature score (Rad_score) was calculated for each lesion using a linear combination of selected features and feature coefficients; these features were weighted with their respective coefficients. The performance of the prediction model was measured by calculating the area under the receiver-operating-characteristic curve (AUC) in the validation cohort (26, 27).

Univariate and multivariate logistic regression analysis were used to identify independent predictors of complete ablation in patients with pulmonary malignancy, including clinical predictors and the rad-score of the training cohort. Then a CT-based radiomics nomogram integrating the radiomics signature and clinical predictors was constructed based on the result of the multivariate logistic regression analysis, which was used to predict the complete ablation of pulmonary malignancies.

### Clinical Application

In the validation cohort, decision curve analysis (DCA) was used to compare the net benefit from the application of the clinical model, the radiomics signature model and combined nomogram model at various threshold probabilities to select the optimal model for individual prediction of the efficacy of thermal ablation for pulmonary malignancies.

### Statistical Analysis

All statistical analyses were performed using R statistical software (R version 3.5.2). Univariate logistic regression analyses were used to assess the associations between clinical risk factors and ablation outcomes. To determine potential associations between variables and ablative efficacy, we compared continuous variables using independent samples t-tests, non-parametric Wilcox test or the Mann–Whitney U test. We analyzed categorical variables using the chi-squared test or Fisher’s exact test. The stepwise logistic regression model was applied for multivariate analysis to identify independent predictors among a combination of factors. Receiver operating characteristic (ROC) curves and the areas under the curves (AUCs) were used to evaluate the performance of predictive model. The AUCs among these models were compared using DeLong’s method. The sensitivity, specificity and positive and negative predictive values were calculated to evaluate the diagnostic performance. The clinical utility of an individual predictive model was evaluated by a decision curve analysis that quantified the net benefits of the two cohorts at various threshold probabilities. A two-tailed analysis was used, and P < 0.05 was considered statistically significant.

## Results

### Patient Characteristics

A total of 104 lesions were detected in 92 patients in our study. Of these, one lesion was detected in 83 patients, two in 7 patients and three in 2 patients. In addition, 38 lesions were confirmed incomplete ablation by enhanced chest CT examination, while 66 were complete ablation. Twenty-seven incomplete and 47 complete ablation lesions were grouped in the training set, while 11 incomplete and 19 complete ablation lesions in the validation set. We applied chi-square or Fisher’s exact test for categorical variables and independent *t* test or Mann–Whitney *U* test for continuous variables to examine the differences in baseline variables between the training and validation sets. Except for the longest, shortest nodule diameter and ratio of long diameter to short diameter, no significant differences were observed in terms of other clinical and CT characteristics between the training and validation data sets. The patient baseline characteristics in two sets are shown **in** **Table 1** and **Supplementary Table 1**. **Supplementary Figure 1** illustrates the work flowchart of the study.

### Inter-Observer and Intra-Observer Reproducibility of Radiomics Feature Extraction

The intra-observer intraclass correlation coefficient ranged from 0.827 to 0.934 and the intraclass correlation coefficient between the two observers ranged from 0.783 to 0.905. The results demonstrated good reproducibility of feature extraction within and between observers.

### Selection of Radiomics Features and Establishment of Radiomics Signature

**Figure 2:**
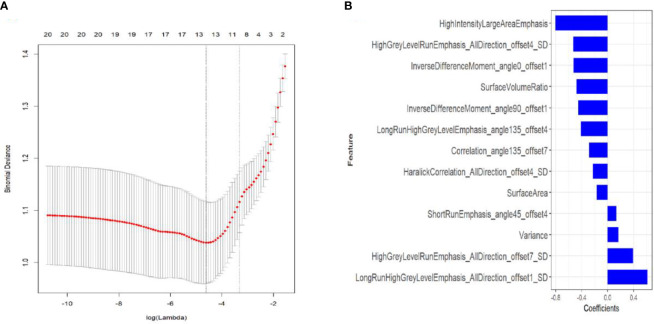
Textural feature selection using least absolute shrinkage and selection operator (LASSO) binary logistic regression. **(A)** Tuning parameters (λ) for the LASSO model were selected by 10-fold cross-validation using the minimum criteria. Partial likelihood deviance was plotted against log (λ). The dotted vertical lines correspond to the optimal values according to the minimum criteria and 1-SE criterion. The 10 features with the smallest binomial deviance were selected. **(B)** LASSO coefficient profiles of texture features. Vertical lines correspond to the values selected by 10-fold cross-validation of the log (λ) sequence; the 10 non-zero coefficients are indicated.

A total of 396 radiomics features were extracted from CT lung window images during pulmonary malignancy ablation by MRMR selection; 20 features were retained following the exclusion of redundant and irrelevant features. Then, LASSO was used to select the optimized feature subset to establish the radiomics signature. Finally, the 13 most valuable variables and their coefficients were retained (**Figure 2**). The values of 13 features were input into the formula, and the Rad-score was obtained to reflect the total ablation value.

The resulting formula was as follows:

Rad-score = -0.525*InverseDifferenceMoment_angle0_offset1+-0.803*HighIntensityLargeAreaEmphasis+0.614*LongRunHighGreyLevelEmphasis_AllDirection_offset1_SD+-0.529*HighGreyLevelRunEmphasis_AllDirection_offset4_SD+-0.481*SurfaceVolumeRatio+-0.227*HaralickCorrelation_AllDirection_offset4_SD+0.132*ShortRunEmphasis_angle45_offset4+-0.454*InverseDifferenceMoment_angle90_offset1+-0.411*LongRunHighGreyLevelEmphasis_angle135_offset4+-0.287*Correlation_angle135_offset7+-0.169*SurfaceArea+0.392*HighGreyLevelRunEmphasis_AllDirection_offset7_SD+0.166*Variance + 0.315).

**Figure 3:**
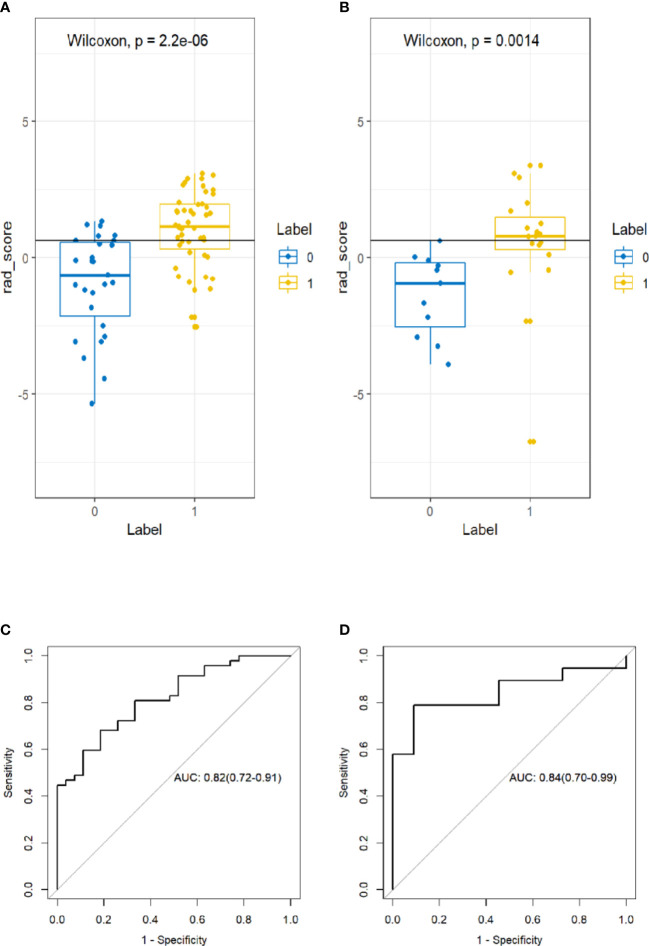
Box plot showing the rad-score distribution of incomplete ablation and complete ablation on training and validation cohorts. p-value from Wilcoxon Rank-Sum test **(A, B)**. Receiver operator characteristic (ROC) curves (training and validation cohorts) **(C, D)**. The prediction performance of the ROC curves for radiomics signature for training and validation cohorts.

A significant difference was found in the Rad-score between incomplete and complete ablation groups in the training set [median -0.60 (IQR, -2.20, 0.60) vs. median 1.10 (IQR 0.30, 2.00); p < 0.001], which was then confirmed in the validation set [median -0.90 (IQR, -2.50, 0.20) vs. median 0.80 (IQR 0.30, 1.50); p = 0.002]. The Rad-score predicting the complete ablation yielded a C-index of 0.82 (95% CI, 0.72–0.91) in the training set, and 0.84 (95% CI, 0.70–0.99) in the validation set. The AUC of the combined radiographic–radiomics model was 0.88 (95% CI, 0.80–0.96) in the training set, and 0.87 (95% CI, 0.71–1.00) in the validation set (**Figure 3**).

### Construction of the Radiomics Nomogram

**Figure 4:**
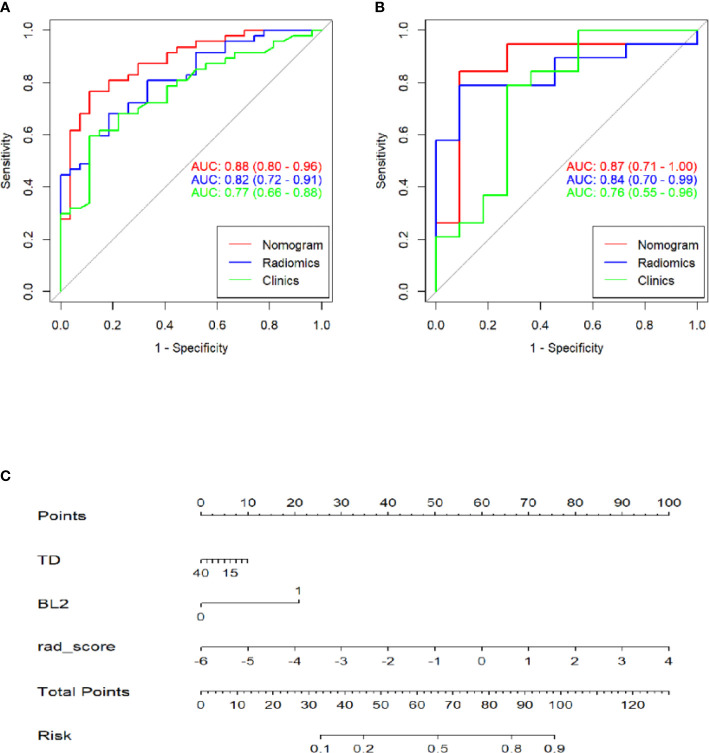
Receiver operating characteristic (ROC) curves of the clinics, radiomics and combinations of computed tomography (CT)-based radiomics signatures used to discriminate between complete and incomplete ablation of pulmonary malignancies in the training and validation cohorts **(A, B)**. Radiomics nomogram **(C)** used to discriminate complete and incomplete ablation of pulmonary malignancies. The nomogram was based on the training cohort; the rad-scores are shown. Initially, vertical lines were drawn at the rad-score values to determine the values of the points. The final point value was the sum of those of the two points. Finally, a vertical line was drawn at the total point value to determine the probability of complete pulmonary malignancy ablation.

A multivariate logistic regression analysis using backward stepwise selection identified that the Rad-score, BL2, and TD were statistically significant independent differentiators of complete ablation from incomplete ablation (**Table 2**), which were incorporated to develop the nomogram (**Figure 4**).

**Table 2:** Clinically significant factors and independent predictors.

Characteristic	Univariate Logistic Regression		Multivariate Logistic Regression	
	OR (95% CI)	p	OR (95% CI)	p
LD	0.90 (0.83;0.98)	0.011	–	–
TD	0.86( 0.78;0.95)	0.004	0.90 (0.81;1.00)	0.0479
BL2	0.77 (2.00;28.97),	0.003	5.21 (1.30;20.85)	0.0195

OR, odd ratio; TD Nodule shortest diameter, LD Nodule longest diameter, BL2 The largest vessel diameter within 1cm around the nodules was 2 mm.

### Performance of the Radiomics Nomogram in the Training Set and Validation Set

**Figure 5:**
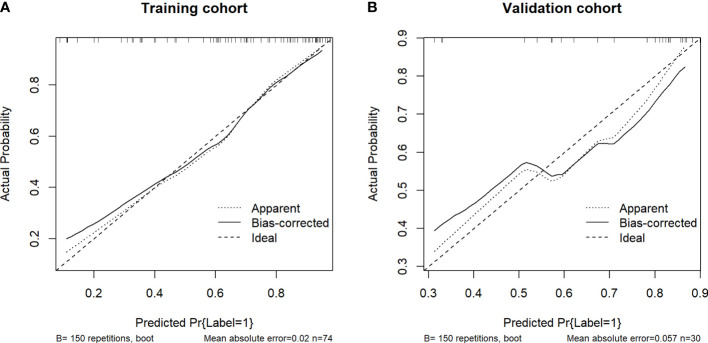
Calibration curves of the nomograms of the training **(A)** and validation **(B)** cohorts. The diagonal dotted lines represent the ideal predictions; the solid lines represent nomogram performance. A closer fit to the diagonal line indicates more accurate prediction.

**Figure 6:**
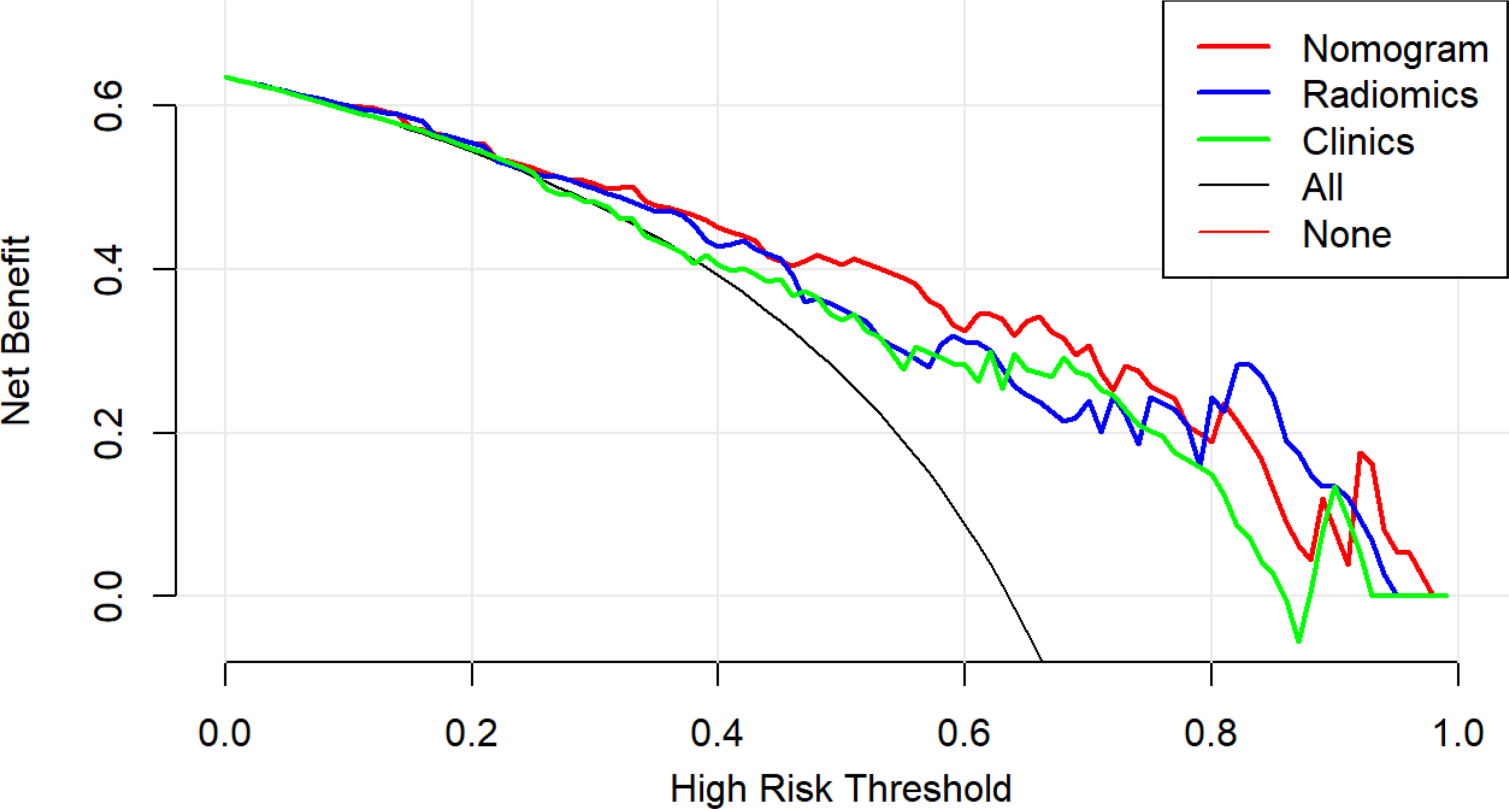
Decision curve analysis (DCA) results for the three discrimination models. The Y-axis represents the net benefit, calculated by summing the benefits (true-positives) and subtracting the weighted harm (i.e., deleting false-positives). The optimal method for feature selection is that with the highest net benefit.

**Table 3** shows the sensitivity and specificity of the three models in the training and validation cohorts, indicating that the radiometric nomograms had good discriminant efficiency. **Figure 5** shows the calibration curve of the radiomics nomogram. The correction curve showed a good correction effect in the training cohort. The validation cohort confirmed favorable calibration of the radiomics nomogram. Decision curve analysis was used to evaluate the clinical usefulness of the clinical model, the radiomics signature model and combined nomogram model in the validation cohort (**Figure 6**). If the threshold probability of clinical decision is between 0.0 and 1.0, it is more advantageous to predict complete ablation of pulmonary malignancies using the radiomics nomogram. In addition, the clinical–radiomics nomogram to predict complete pulmonary malignancy ablation provided more net benefits than the radiomics prediction model alone or clinical prediction model alone. The results of Delong test suggested that in the training cohort, there was a significant difference in AUC between the combined model and the clinical model (p=0.02), combined model and the radiomics model (p=0.03).There was no significant difference in AUC between the radiomics model and clinical model (p= 0.38).

**Table 3:** Predictive performance of three prediction models for the training and validation sets.

Training cohort	AUC	95%CI	Sensitivity	Specificity	Accuracy	PPV	NPV
Clinical prediction model	0.77*	0.66-0.88	0.681	0.815	0.730	0.865	0.595
Radiomics signature	0.82*	0.72-0.91	0.596	0.889	0.703	0.903	0.558
Clinical–radiomics nomogram	0.88	0.80-0.96	0.811	0.889	0.766	0.923	0.686
Validation cohort	AUC	95%CI	Sensitivity	Specificity	Accuracy	PPV	NPV
Clinical prediction model	0.76	0.55-0.96	0.579	1.00	0.733	1.00	0.579
Radiomics signature	0.84	0.70-0.99	0.369	0.819	0.5339	0.778	0.429
Clinical–radiomics nomogram	0.87	0.71-1.00	0.941	0.769	0.867	0.842	0.909

CI, confidence interval; AUC, area under the curve; PPV, positive value; NPV, negative predictive value.

*P < 0.05 indicates statistically significant differences between AUCs of clinical prediction model (or radiomics model) and clinical–radiomics model with DeLong’s test.

## Discussion

In our study, we developed and validated a CT-based radiomics nomogram which combined the radiomics signature with independent clinical predictors to predict the outcome of thermal ablation for lung malignancies. In the radiomics prediction model and clinical prediction models, the training and validation cohorts showed good differentiation. The discrimination ability of the clinical–radiomics nomogram was superior to that of the other two models, which provides important information for intraoperative decision-making.

The “heat sink effect” has a substantial impact on thermal ablation of pulmonary malignancies. Our study demonstrated that the largest diameter of blood vessels within 1 cm from the lesion was 2 mm; this was an independent predictor of intraoperative complete ablation. When the largest diameter of the blood vessels around the lesion is 2 mm, the possibility of complete ablation of the lesion is high, which has important clinical significance. A previous study showed that the ablation volume would decrease when the vessel diameter was larger than 3 mm (28). This finding is similar to ours in that when the largest diameter of the blood vessels adjacent to the lesion is close to 3mm, it can increase the possibility of incomplete ablation (29). The findings provide important insights for clinicians during intraoperative decision-making. Giraud et al. found that 95% of lung malignancies had microinvasion. In general, the invasive depth of adenocarcinoma is about 8mm, and that of squamous cell carcinoma is about 6mm (30). Therefore, we should obtain a 10-mm safe ablation zone around the lesion to ensure coagulation necrosis of all tumor cells during thermal ablation (13). However, during the operation, inflammatory exudation, hyperemia, and acute bleeding around the ablation foci also present as ground glass shadow; thus, visual assessment of complete ablation depends on the knowledge and experience of the clinicians. The judgment of complete ablation has high subjectivity and low accuracy. Simple visual analysis cannot detect deeper lesions intraoperatively; thus, it cannot meet the requirements for precision medicine and individualized treatment.

Imaging omics reflects tumor heterogeneity and provides pathophysiological information regarding tumor lesions (25). The coagulation necrosis of tumor cells after ablation of pulmonary malignancies involves unique pathophysiological changes. Therefore, we established a high-throughput database of CT images during thermal ablation of pulmonary malignancies, then used the mRMR and LASSO algorithm to screen and select radiomics features, and finally obtained 13 potential predictors. Among these features, the characteristics of tumor shape (surface area, surface to volume ratio) and gray level co-occurrence matrix (correlation and inverse difference moment) were more negatively correlated with complete ablation. Larger lesion size was associated with greater surface area and greater possibility of incomplete ablation, consistent with our clinical experience. The surface-to-volume ratio and gray level co-occurrence matrix features reflect the complexity and heterogeneity of tumor internal structure; the higher the value of these two indexes, the more complex and heterogeneous the tumor is (31, 32). The theoretical basis is presumably that the less coagulative necrosis of tumor cells after thermal ablation, the more tumor cells remain. Due to the existence of heterogeneity of tumor cells, the whole ablation focus shows great heterogeneity. Complete ablation is positive correlated with gray trip matrix characteristics (short run emphasis, high gray level run emphasis, and long run high gray level emphasis), implying that higher image intensity and smooth texture are indicators of lower heterogeneity of ablation lesions (33, 34).

To the best of our knowledge, this is the first radiological combined model based on CT images during pulmonary lesion ablation, which can predict the complete ablation of pulmonary lesions and serve as a tool to help clinicians make informed decisions during the operation. This study has several strengths. Firstly, the prediction model is developed based on a multicenter study and internal validated. Secondly, through multivariate analysis using clinical and imaging data, we firstly found that the largest diameter of blood vessels within 1 cm from the lesion being 2 mm is a significant predictor for complete ablation of pulmonary lesions. Third, mRMR and LASSO methods were used as feature selection and dimension reduction schemes for high-throughput data of intraoperative CT images, and a radiomics-based prediction model is developed based on these features.

The AUC value, sensitivity, specificity, and accuracy were high in the training and validation cohorts, suggesting that the radiomics-based prediction model has a good predictive performance. The predictive efficiency of the radiomics model based on CT radiomics features was better than the clinical prediction model based on clinical risk factors in the training and validation cohorts. Moreover, the radiological combined model demonstrated the best calibration and discrimination performance than both the clinical prediction model and the radiomics prediction model, demonstrating its superiority in clinical practice.

This study has some limitations. Firstly, the number of patients and lesions were small. To further validate the robustness and repeatability of our prediction model, larger scale prospective cohort studies involving patients from some more other centers are warranted for external validation. Secondly, we suspected that different machines and different CT scanning parameters might influence the radiomics features and analyses of prediction model (35). However, the actual results were good. Whether this finding is generalizable to other hospitals using other CT scanner parameters requires further prospective and multicenter studies. Thirdly, diagnostic biopsy is not routinely performed for lung metastases. Therefore, newly discovered or enlarged focal lung tumors within a short period of time were regarded as lung metastases because of previously identified primary cancer in these patients.

In conclusion, our study shows that a radiological combined prediction model based on CT lung window images can be a good predictor of complete ablation in patients with pulmonary malignancies, which can aid in clinical decision-making during thermal ablation of pulmonary malignancies. Furthermore, based on the results of our study and future validation using larger samples, maybe we will translate the prediction model into a visual and operational WeChat Mini Program, which can be used for real-time evaluation of the complete ablation of pulmonary lesions during operation.

## Data Availability Statement

The original data could be provided on reasonable request from the authors. Requests to access these datasets should be directed to GZ, zgz_0007@163.com.

## Ethics Statement

The studies involving human participants were reviewed and approved by the ethics committees of Cancer Hospital of the University of Chinese Academy of Sciences and Huzhou Central Hospital. Written informed consent for participation was not required for this study in accordance with the national legislation and the institutional requirements.

## Author Contributions

GZ and YW had full access to all of the data in the study and took responsibility for the integrity of the data and the accuracy of the data analysis. GZ and HY are co-first authors of this article. Study concept and design: GZ, ZM, and GS. Acquisition, analysis, or interpretation of data: GZ, HY, XZ, JL, JZ, YX, YZ, and YW. Drafting of the manuscript: GZ, HY, and ZM. Critical revision of the manuscript for important intellectual content: All authors. Statistical analysis: GZ, HY, and YW. Administrative, technical, or material support: All authors. Study supervision: GS. All authors contributed to the article and approved the submitted version.

## Funding

This work was sponsored by Quzhou Technology Project (2021038).

## Conflict of Interest

Author YW was employed by General Electric (GE) Healthcare.

The remaining authors declare that the research was conducted in the absence of any commercial or financial relationships that could be construed as a potential conflict of interest.

The reviewer SY declared a shared parent affiliation with one of the authors, ZM, to the handling editor at time of review.

## Publisher’s Note

All claims expressed in this article are solely those of the authors and do not necessarily represent those of their affiliated organizations, or those of the publisher, the editors and the reviewers. Any product that may be evaluated in this article, or claim that may be made by its manufacturer, is not guaranteed or endorsed by the publisher.

## Supplementary Material

Supplementary Figure 1 The work flowchart of the study.
