# Supplementary figures and images for: A CT-Based Radiomics Nomogram to Predict Complete Ablation of Pulmonary Malignancy: A Multicenter Study
Source: Front Oncol. 2022 Feb 10;12:841678. doi: 10.3389/fonc.2022.841678 (PMC8866938; doi:10.3389/fonc.2022.841678)

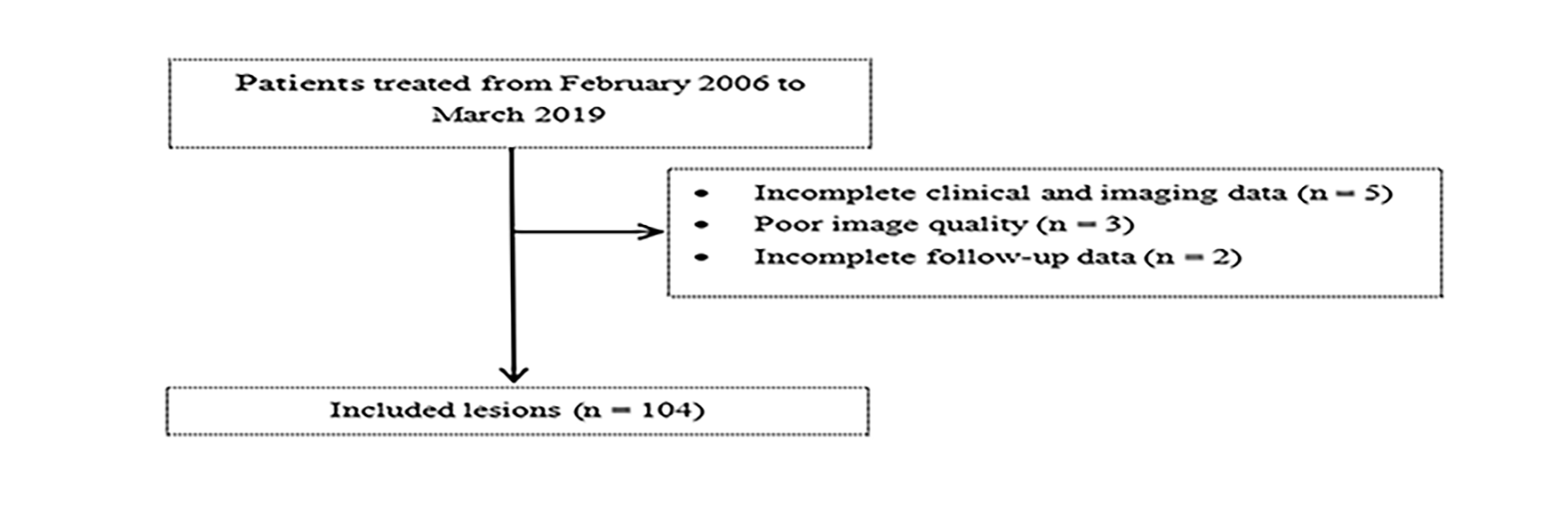

Supplement: Supplementary Figure 1 — The work flowchart of the study. [file Image_1.tif]
