# Supplementary material for: A CT-Based Radiomics Nomogram to Predict Complete Ablation of Pulmonary Malignancy: A Multicenter Study
Source: Front Oncol. 2022 Feb 10;12:841678. doi: 10.3389/fonc.2022.841678 (PMC8866938; doi:10.3389/fonc.2022.841678)
Supplement: Supplementary file 2 [file Table_1.docx]

**Supplementary Table 1.** Variables of patients in incomplete and complete ablation groups included in the training set and validation set

| **Variables** | **Data** | **Incomplete ablation (n=38)** | **Complete ablation (n=66)** | **p value** |
| --- | --- | --- | --- | --- |
| Age (years) | 60.5 ± 10.1 | 59.2 ± 9.5 | 61.3 ± 10.5 | 0.29 |
| Gender |  |  |  | 0.026 |
| Male | 74 (71.2%) | 32 (84.2%) | 42 (63.6%) |  |
| Female | 30 (28.8%) | 6 (15.8%) | 24 (36.4%) |  |
| Treatment options |  |  |  | 0.945 |
| Radio-frequency ablation | 90 (86.5%) | 33 (86.8%) | 57 (86.4%) |  |
| Microwave ablation | 14 (13.5%) | 5 (13.2%) | 9 (13.6%) |  |
| Nodule Shape |  |  |  |  |
| Class round | 84 (80.8%) | 29 (76.3%) | 55 (83.3%) |  |
| Irregularly shaped | 20 (19.2%) | 9 (23.7%) | 11 (16.7%) |  |
| LD | 18.5 ± 8.1 | 23.0 ± 10.1 | 15.9 ± 5.3 | <0.001 |
| TD | 14.6 ± 6.1 | 17.9 ± 7.0 | 12.7 ± 0.49 | <0.001 |
| LD/TD | 1.3 ± 0.3 | 1.3 ± 0.3 | 1.3 ± 0.3 | 0.648 |
| Blood vessel diameter |  |  |  | 0.051 |
| 1mm | 39 (37.5%) | 10 (26.3%) | 29 (43.9%) |  |
| 2mm | 37 (35.6%) | 13 (34.2%) | 24 (36.4%) |  |
| 3mm | 20 (19.2%) | 9 (23.7%) | 11 (16.7%) |  |
| 4mm | 8 (7.7%) | 6 (15.8%) | 2 (3.0%) |  |
| Bronchial diameter |  |  |  | <0.001 |
| 1mm | 17 (16.3%) | 5 (13.2%) | 12 (18.2%) |  |
| 2mm | 35 (33.7%) | 5 (13.2%) | 30 (45.5%) |  |
| 3mm | 29 (27.9%) | 12 (31.6%) | 17 (25.8%) |  |
| 4mm | 11 (10.6%) | 6 (15.8%) | 5 (7.6%) |  |
| 5mm | 9 (8.7%) | 7 (18.4%) | 2 (3.0%) |  |
| 6mm | 3 (2.9%) | 3 (7.9%) | 0 (0.0%) |  |
| Tumor location |  |  |  |  |
| The right lung |  |  |  | 0.017 |
| Superior lobe | 26 (25.0%) | 11 (28.9%) | 15 (22.7%) |  |
| Middle lobe | 9 (8.7%) | 1 (2.6%) | 8 (12.1%) |  |
| Inferior lobe | 23 (22.1%) | 9 (23.7%) | 14 (21.2%) |  |
| The left lung |  |  |  | 0.142 |
| Superior lobe | 18 (17.3%) | 9 (23.7%) | 9 (13.6%) |  |
| Inferior lobe | 28 (26.9%) | 8 (21.1%) | 20 (30.3%) |  |
| Distance from nodule to pleura | 12.8 ± 10.0 | 11.9 ± 9.6 | 13.3 ± 10.2 | 0.646 |

Ages, TD and LD shown as mean ± standard deviation; Gender, treatment options, nodule shape are the number of patients with the percentage in parentheses.

*P* value is derived from the univariable association analyses between the training set and validation set.

TD, transverse diameter of the largest layer of the nodule; LD, the longitudinal diameter of the largest layer of the nodule; LD/TD, ratio of the longitudinal to transverse diameter; bronchial diameter, the largest vascular diameter within 1cm around the nodule; Blood vessel diameter, the largest diameter bronchus within 1cm around the nodule.

| **Supplementary Table 2**. Parameters of the thermal ablation equipment | | | |
| --- | --- | --- | --- |
|  | Ablation needle | Working frequency | Working power |
| Cool-Trip RFA system | ACT-1530 or ACT-1520 | 480 kHz | 0~200 W |
| KY-2000 system | KY-2450B-1 | 2450 MHz | 0~150 W |

| Setting | GE Lightspeed | Somatom Sensation 16 | Philips Brilliance CT 16 |
| --- | --- | --- | --- |
| Tube voltage(kVp) | 120 | 120 | 120 |
| Tube current(mA) | 150 | 110 | 200 |
| Pitch | 1.0 | 1.0 | 0.8 |
| Rotation time(s/rot) | 0.75 | 0.6 | 0.75 |
| SFOV(cm) | 50 | 50 | 50 |
| Slice thickness(mm) | 5 | 5 | 3 |
| Slice interval(mm) | 5 | 5 | 3 |

**Supplementary Table 3.** CT scanning parameters
